# Hyaluronic acid-coated chitosan nanoparticles as targeted-carrier of tamoxifen against MCF7 and TMX-resistant MCF7 cells

**DOI:** 10.1007/s10856-022-06647-6

**Published:** 2022-02-14

**Authors:** Fariba Nokhodi, Mehdi Nekoei, Mohammad Taghi Goodarzi

**Affiliations:** 1 Department of Chemistry, Shahrood Branch, Islamic Azad University, Shahrood, Iran; 2 Department of Biochemistry, Shahrood Branch, Islamic Azad University, Shahrood, Iran

## Abstract

Tamoxifen (TMX) is used to treat hormone-receptor-positive breast cancers at early stages. This research aimed to assess the potential of NPs in targeted delivery of TMX against MCF7 and TMX-resistant MCF7 breast cancer cell lines. For this purpose, a targeted delivery system including chitosan NPs coated with hyaluronic acid (HA-CS NPs) was created and examined in vitro. Chitosan NPs were first fabricated and loaded with TMX using the ionic-gelation method to prepare a drug-delivery system. Then, TMX-loaded CS NPs were coated by crosslinking the amino groups of chitosan to the carboxylic group of hyaluronic acid. The developed TMX delivery system was then optimized and characterized for particle fabrication, drug release, and targeting against cancer cells. The HA-CS particle size was 210 nm and its zeta potential was +25 mv. The encapsulation efficiency of TMX in NPs was 55%. TMX released from the NPs in acidic pH (5–6) was higher than the physiological pH (7.4). The cytotoxic effect of TMX-loaded HA-CS NPs on MCF7 and TMX-resistant MCF7 cells was significantly higher than TMX-loaded CS NPs and free drug. The findings confirmed the significant suppressive impact of TMX-loaded HA-CS NPs on MCF7 and TMX-resistant MCF7 cancer cells compared to the TMX-loaded CS NPs and free TMX.

**Figure:**
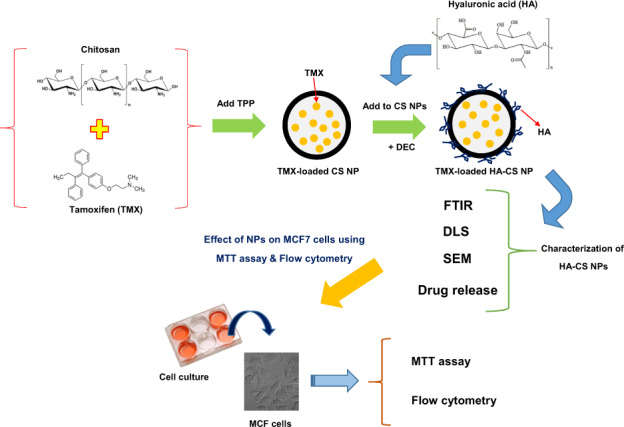
Graphical abstract

Graphical abstract

## Introduction

The incidence of cancer as the second biggest killer globally in the recent millennium highlights the importance of excogitating efficient therapies to conquer the increasing prevalence of cancer [1]. As an example, only in 2015, 8.8 million died of cancer around the world which makes it one-sixth of deaths according to the World Health Organization (WHO) [2]. One of the most frequent cancer types and the first cause of death among cancers in women is breast cancer; however, it is not limited to women. Fortunately, non-metastatic breast cancers are curable in around 70–80% of cases, especially at the early stages of the disease [3]. As a heterogeneous disease, several molecular malfunctions can result in breast cancer. Point mutations in the human epidermal growth factor receptor 2 (HER2) gene, aberrations in the expression of estrogen (ERα and/or ERβ) and progesterone receptors (PR), and/or harmful mutations in breast cancer gene (BRCA1 and/or BRCA2) are the most recognized reasons underlying the breast cancer incidence [4, 5]. Therefore, not only in etiology, breast cancer shows multiple pathologies including its prognosis which can vary from clinical invasive conditions to end-stages [6]. Increasing the total population and ascending breast cancer incidences has reached its estimated prevalence of up to 3.2 million in 2050 that necessitates serious attention to novel methods for preventing and treating this malignancy [7]. The current common therapeutic strategies for curing breast cancer include surgery, radiotherapy, chemotherapy, immunotherapy, hormone therapy, cell therapy, etc. [8], but all of these methods, despite showing different levels of effectiveness, have different side effects. One of the main reasons for emerging side effects is the non-targeted nature of the conventional methods of cancer therapy that cause harmfulness in healthy tissues [9]. The other reason for the inefficiency of conventional cancer therapy methods, especially in metastatic cancers, is the development of drug resistance [10]. Targeted therapy is a novel strategy that has been suggested and broadly studied recently to overcome the aforementioned limitations of conventional cancer therapies including the breast cancer.

The hormone requiring the character of hormone-receptor-positive breast cancers for female hormones (i.e., estrogen and/or progesterone) [11] has brought up the idea of hormone therapies that include using hormone-receptor modulators of which tamoxifen (TMX) is the oldest one. Over 40 years, TMX has been applied for treating both locally advanced and metastatic hormone-receptor-positive breast cancers at early stages [12, 13]. The most frequent obstacle about TMX-therapy is drug-resistance development in patients after the initial five to ten years of receiving this medicine [14]. Other side effects, including blood clots in the large veins and lungs (pulmonary emboli), bone loss, uterus cancer emergence, cataracts, and stroke, can be also associated with this drug [15]. Among several therapeutic strategies tested for killing cancerous cells with fewer side effects, using nanoparticles (NPs) for carrying the drugs to a specific favorite tissue has been one of the most recognized methods in terms of effectiveness, stability, and applicability [8]. NPs have shown to be highly advantageous carriers for drug delivery since they have more plasma half-life, more targetable biodistribution profile, and surface functionalization ability [16, 17]. Chitosan (CS) is one of the popular nanocarriers - a linear polysaccharide that is widely distributed in nature and used in laboratories by researchers for developing drug-delivery systems. CS is a biocompatible, biodegradable, and modifiable biopolymer with regards to being safe for pharmaceutics applications in humans [18]. On the other hand, the relatively high positive charge density of chitosan, along with its rapid agglomeration at physiological pH as well as the absorption of nonspecific proteins, can lead to increased cytotoxicity and nonspecific uptake of CS NPs by cells [19]. Polymer grafting is a method by which the different blocks are attached to the backbone of a natural polymer as side chains to improve their properties such as solubility, reactivity, and etc, which can have many applications in drug-delivery systems [20, 21]. Accordingly, the biochemical and mechanical properties of chitosan are improvable by joining some macromolecules or functional groups to its molecular structure. A potential solution to overcome these drawbacks is complexation/decoration of CS NPs with anionic macromolecules, such as alginate, glycosaminoglycans (GAGs), poly(γ)-glutamic acid (PGA), heparin/heparin sulfate, and hyaluronic acid (HA) [19]. According to the studies, HA is one of the most popular macromolecular components that nanoparticles such as CS can be coated with [22–24]. The advantages of HA coating are resistance to protein adsorption, which is associated with longer circulation time in the blood and thus provides a relative “hiding” property for the nanoparticle. It has been shown that uptake of HA-coated CS nanoparticles by macrophage is 2 to 3 times slower than uncoated nanoparticles. Also, its affinity to bind to CD44 antigen is another advantage of using HA [22].

In general, the modification of NPs can be aimed at increasing their targetedness in either a passive or active form of drug delivery. Passive-enhancement of drug delivery is the state of increasing the penetration of NP to the target site through enhanced permeability and retention (EPR) effect. Whereas, active-enhancement of drug delivery by NPs contains structural modifications and functionalization that lead to more targeting capability [25]. A famous compound that is widely used for developing active-targeted NPs for drug delivery purposes is hyaluronic acid (HA) because of its water-solubility, biodegradability, biocompatibility, low toxicity for cells, and non-immunogenicity in the body [26, 27]. HA is a natural polysaccharide that shows specificity to the integral membrane glycoprotein CD44 on the surface of eukaryotic cells. Some types of tumor cells overexpress CD44 on their surface that facilitates targeting them by increasing the concentration of NPs in the tumor tissues. Hence, HA is extensively utilized in designing drug-delivery products as a targeting component with the final object of decreasing the amount of drug consumption and adverse effects on the healthy tissues [17, 28, 29].

In the current study, HA-coated CS NPs were fabricated and applied for targeted delivery of tamoxifen against CD44-overexpression MCF7 and TMX-resistant MCF7 breast cancer cell lines. For this purpose, the specificity of HA-CSNPs to these cell lines and anti-cancer properties of TMX-loaded HA-CS NPs were compared to the free drug. However, in recent years, various studies have been performed on the targeted release of tamoxifen against breast cancer cells using nanocarriers [30]. In this study, for the first time, we evaluated HA-CS NPs for encapsulation and targeted release of tamoxifen.

## Materials and methods

### Materials

Chitosan (CS) [low molecular weight (MW = 50–160 kDa), 85% deacetylation degree], hyaluronic acid (HA) (MW = 100 kDa), and tamoxifen (TMX), as well as tripolyphosphate (TPP), 3-(4, 5-dimethylthiazol-2-yl)-2, 5-diphenyltetrazolium bromide (MTT), and dimethyl sulfoxide (DMSO) were purchased from Sigma-Aldrich, USA. Glacial acetic acid and 1-ethyl-3-(3-dimethylaminopropyl) carbodiimide (EDC) were obtained from Merck, Germany. Fetal bovine serum (FBS) and Dulbecco’s Modified Eagle’s Medium (DMEM) were supplied from Gibco, USA. CD44-overexpressing MCF7 breast cancer cell lines were bought from Iranian Biological Resource Center (IBRC), Tehran, Iran, which were confirmed for overexpression of the CD44 antigen. Human fibroblast (HF) (used as normal control cells in the following experiments) were purchased from the National Cell Bank of Iran (NCBI), Tehran, Iran. Double-distilled deionized water (DW) from Zolal, Iran, was used in all experiments.

### Methods

#### Preparation of TMX-loaded chitosan nanoparticle (CS NPs)

TMX-loaded CS NPs were created using Calvo et al.’s version of the ionic-gelation method [31]. Different concentrations of CS solution were prepared by dissolving CS in the aqueous acetic acid (1% v/v) and left on a magnet stirrer overnight at room temperature to be completely dissolved. TPP was dissolved in double-distilled deionized water with a concentration of 1 mg/ml. CS and TPP solutions were passed through a 0.45 µm pore size filter. After that, different amounts of tamoxifen (TMX) were added to the CS solution (Table 1). Since TMX is better solved in organic solvents such as DMSO and ethanol, and sparingly soluble in aqueous buffers, it was first dissolved in a solution of ethanol/PBS (pH 7.2) and then diluted with the aqueous buffer for maximum solubility. TMX was solved in ethanol/PBS up to approximately 0.5 mg/ml (Product Criteria, Sigma). Then, the TPP solution was added dropwise to the chitosan solution at different CS:TPP mass ratios to produce CS NPs. The mixtures were left on a magnetic stirrer at 600 rpm for 30 min at ambient temperature. During NPs formation, a cloudy solution appeared. Then, NPs were separated from the solution by centrifuging (18,000 rpm) at 10 °C for 30 min. For removing any probable free TMX left on the surface of NPs and unreacted polymers, the collected NPs were washed trice with DDDIW and acetic acid (2% v/v) and centrifuged (18,000 rpm) at 10 °C for 10 min. The obtained supernatant was stored to be used for measuring the encapsulation efficiency (EE%) and the deposited NPs were stored at 4 °C for further analyses.

**Table 1:** Independent and dependent variables used in Box–Behnken design

		Level		
Code	Independent variables (Factors)	Low (−1	Middle (0)	High (+1)
A	Chitosan concentration (mg/ml)	0.5	1	1.5
B	Chitosan/TPP (mass)	3	6	9
C	TMX/Chitosan (mass)	0.5	1	1.5
	Dependent Variables (Responses)			
R_1_	Encapsulation efficiency (%)	Maximum		
R_2_	Particles size (μm)	Minimum		

#### Coating chitosan nanoparticles with hyaluronic acid

Bonding the hyaluronic acid carboxylic group to the chitosan amino group was performed using EDC as the coupling factor [32]. The steps were taken according to Taghipour-Sabzevar et al.‘s [17]. Briefly, the optimized NPs in terms of EE% and size (according to the Design of Experiment [DOE] or Design-Expert software) were suspended in PBS (pH 7.4). Accordingly, EDC was added to the HA solution and then the mixture was added dropwise to CS NPs on a magnetic stirrer at 400 rpm in the environment temperature for 1 h. The obtained solution was centrifuged (18,000 rpm) at 10 °C for 30 min to separate NPs. The deposit of nanoparticles was washed with DW to remove any probable unreacted materials around and on NPs.

#### Experimental design

An experiment was designed using the response surface method (RSM) to minimize the particle size and maximize the encapsulation efficiency or EE%. Accordingly, a Box–Behnken was designed with three variables: 1) CS concentration (mg/ml); 2) mass CS:TPP ratio, and 3) mass TMX:CS ratio, at 3 levels with 4 center points and a total of 16 runs to optimize the dependent variables. Details of the experimental design are represented in Tables 1 and 2. The observed data were analyzed for each response by DESIGN-EXPERT 8.1 software and the fitting of the experimental data to the regression model was checked. The influence of independent variables and their interaction were evaluated by Analysis of Variance (ANOVA). *P* value was considered <0.05 for statistical significance in all analyses.

**Table 2:** Box–Behnken design matrix with three independent variables at three levels and the observed responses

Run	CS Conc.(mg/ml)	CS/TPP	Drug/CS	EE (%)	Diameter size (nm)
1	1.0	3.0	0.5	13.9 ± 1.2	148 ± 7.9
2	1.5	6.0	1.5	35.8 ± 3.4	131 ± 10.2
3	1.0	3.0	1.5	61.3 ± 3.4	159 ± 20.1
4	1.5	9.0	1	38.2 ± 1.2	140 ± 11.3
5	1.0	6.0	1	50.7 ± 2.6	147 ± 5.7
6	1.0	9.0	0.5	62.1 ± 2.1	150 ± 14.1
7	0.5	6.0	1.5	43.3 ± 6.1	189 ± 24.1
8	0.5	9.0	1.0	29.5 ± 2.3	235 ± 19.7
9	1.5	3.0	1.0	13.1 ± 1.1	159 ± 15.2
10	1.0	6.0	1.0	48.9 ± 1.2	171 ± 15.2
11	1.0	6.0	1.0	46.4 ± 3.1	130 ± 5.31
12	0.5	6.0	0.5	26.6 ± 3.3	156 ± 9.11
13	1.0	9.0	1.5	24.6 ± 2.9	202 ± 11.1
14	0.5	3.0	1.0	25.7 ± 3.2	184 ± 20.1
15	1.0	6.0	1.0	54.2 ± 3.1	138 ± 19.8
16	1.5	6.0	0.5	29.1 ± 2.1	137 ± 10.1
Optimum	**1.13**	**9**	**0.5**	**60** ± **2.2**	**164** ± **7.3**

Bold entries represent general values which are optimized by related software as optimum values

#### Particle-size determination

The particle size distribution, poly-dispersity index, and zeta potential of the NPs were determined using the dynamic light scattering (DLS) technique by a Zetasizer (Nano–ZS, Malvern Instruments Ltd, Worcestershire, UK). DLS determination was exerted at 25 °C with a 173-degree scattering angle.

#### Measurement of EE%

For EE% measurement, NP suspension was centrifuged and NPs were separated from the medium. The supernatant was collected and its TMX amount was measured by a UV–visible spectrophotometer (Optizen 2120 UV–visible spectrophotometer, Korea) at λ_max_ = 276 nm and compared with the UV absorbance of the corresponding blank NPs of the same composition without TMX. The supernatant TMX content was measured using a calibration curve plotted by the UV absorbance measures of eight pre-defined concentrations of TMX. Then, the total content of the supernatant was evaluated by multiplying the measured concentration and volume of the supernatant. EE% of the TMX was calculated according to Eq. (1):

1 $${\mathrm{EE}}\% = \frac{{Wt - Ws}}{{Wt}} \times {{{\mathrm{ }}}}100$$

In the above equation, EE% shows the encapsulation efficiency of NPs, W_t_ shows the total amount of TMX utilized for NPs preparation, and W_s_ shows the amount of free TMX in the supernatant.

#### Fourier-transform infrared spectroscopy (FTIR)

An FTIR spectroscopy (Shimadzu, IRaffinity-1, Osaka, Japan) technique was used in the range of 400–4000 cm^−1^ to corroborate the TPP/CS bonding and HA/CS NPs coupling. KBr was used for mixing the polymer samples and then they were compressed on a hydraulic press to make pellets.

#### Scanning electron microscopy (SEM)

The NPs’ morphological characterization (aggregation and shape) was performed using the SEM technique (JEOL JEM-1230, Japan). In this regard, NP samples were sonicated and dried in an oven under vacuum conditions for 24 h and coated with a thin layer of gold before being examined.

#### In vitro tamoxifen release studies

For cancer treatment purposes, NPs are designed to enhance the bioavailability of drugs in the tumor microenvironments and minimize its side effects in the normal cells. The microenvironments of tumors are mostly acidic; then, the NPs are preferred to be less released in the physiological pH (7.4) than in the acidic environment around the tumors. For evaluating the in vitro release of tamoxifen from NPs, TMX-loaded samples were mixed with PBS (pH adjusted to be 5 and 7.4) and transferred to a dialysis bag (MWCO 12 kDa). The dialysis bags were submerged in PBS medium (30 ml at a specific pH) and then placed in a bain-marie heated bath at 37 °C and 130 rpm. After the specified time, 1 ml of dialysate was sampled and the remained was disposed. The same volume of fresh PBS was refilled in the Becher. The amount of TMX that was released into the dialysate was measured using a UV–visible spectrophotometer and the standard TMX curve [17, 33].

#### In vitro cytotoxicity studies by MTT assay

The fabricated NPs were examined for their cytotoxicity effect on MCF7 and TMX-resistant MCF7 breast cancer cell lines and HF (as a non-cancerous cell) using MTT assay. For this aim, 5 × 10^3^ cells per well were precultured in a 96-well plate which was incubated at 37 °C for 24 h with 5% CO_2_ and 90% humidity. Thereafter, different concentrations of free TMX, CS NPs, HA-CS NPs, TMX-loaded CS NPs, and TMX-loaded HA-CS NPs (according to the details summarized in Table 3) were added to wells and the plate was incubated for 24 and 48 h. In the control well, cells were incubated in the normal medium. Then, the cell metabolic activity of each well was assessed by adding 20 μl of MTT (5 mg/ml in PBS) and again incubating the plates under the aforementioned conditions. The unreacted dye was removed after 4 h and 100 μl DMSO was added to each well to dissolve formazan crystals in the living cells. The absorbance of each well was read at 570 nm in a 680 microplate reader (Bio-Rad Laboratories, Hercules, USA). The viability of cells was measured in percent by the formula “AT/AC × 100” where AT represents the absorbance of treated cells and AC represents the absorbance of control cells [34]. For precisely comparing the cytotoxicity of every single constituent presenting in the drug release system, all elements, including the empty HA-CS NPs and CS NPs at the corresponding concentration, were examined for their cytotoxicity effect on the cells, separately.

**Table 3:** The concentrations of TMX, CS NPs, TMX-loaded CS NPs, TMX-loaded HA-CS NPs, and HA-CS NPs used in the MTT assay

Concentration	TMX	CS NPs	TMX-loaded CS NPs	TMX-loaded HA-CS NPs	HA-CS NPs
C1 (μg/ml)	3	9.4	12.4	18	15
C2 (μg/ml)	6	18.8	24.8	36	30
C3 (μg/ml)	12	37.6	49.6	72	60
C4 (μg/ml)	24	75.2	99.2	144	124

#### Assessment of apoptosis by flow cytometry

The apoptotic and necrotic effects of the TMX-loaded HA-CS NPs on the MCF7 cells were evaluated using an annexin V/PI staining kit (BD Biosciences, USA) according to the manufacturer’s instructions. In summary, a 6-well plate was cultured with 2 × 10^5^ cells per well and incubated at 37 °C for 24 h in the presence of 5% CO_2_ and 90% humidity. The next day, cells were exposed to different concentrations of TMX-loaded HA-CS NPs for 8 h (Table 3). Then, cells were deposited by centrifugation, washed twice with cold PBS, and mixed with labeling solutions (annexin V-FITC [5 μl] and propidium iodide (PI) [5 μl]). The obtained cell suspensions were first incubated in the dark for 15 min at ambient temperature and then were analyzed by BD (Becton, Dickinson) FACS Calibur Flow Cytometer and FlowJo software (Version 7.6.1, Tree Star Software; CA, USA).

#### Statistical analysis

One-way ANOVA followed by Bonferroni’s post-hoc comparisons tests were performed using SPSS 18.0 (SPSS Inc, Chicago, IL). The results of the three independent experiments were expressed as the mean ± standard deviation (SD). A *p* ≤ 0.05 was considered as the statistical significance.

## Results

### Fabrication and optimization of chitosan nanoparticles

#### Optimization of size

The NP size is highly important for drug delivery in cancer therapy; therefore, in the present study, an optimized state was selected for chitosan (CS), CS:TPP ratio, and TMX:CS ratio according to the NPs size measured by DOE software. The NP size was variable in the range of 130–240 nm (Table 2). Results were checked with the central composite statistical design and all values for 16 runs showed to be fitted to first order, second order, quadratic, and modified quadratic models (Table 4). The *P* values for the modified quadratic model and lack of fit were 0.0059 (significant) and 0.6367 (not significant), respectively, and R^2^ was 0.8625 (near to 1). The used model showed to be the best compared to the same data from the first order, second-order, and quadratic models.

**Table 4:** Results of regression analysis of responses for size & EE%

Responses	Model	*P* value of model	*P* value of lack of fit	R^2^	Adjusted R^2^	Predicted R^2^	Adeq. precision
Size							
	Linear	0.0269	0.3111	0.51	0.3987	0.1124	6.502
	2FI	0.0431	0.3486	0.6786	0.4862	−0.0214	6.334
	Quadratic	0.0199	0.6997	0.8873	0.7506	0.3478	8.913
	Modified Quadratic	0.0105	0.7711	0.9021	0.7674	0.5412	9.508
Encapsulation efficiency (EE%)							
	Linear	0.6972	0.0044	0.1172	−0.1113	−0.6549	2.189
	2FI	0.1315	0.0087	0.6019	0.3406	−0.2548	5.934
	Quadratic	0.0003	0.2332	0.9978	0.9389	0.7289	17.116
	Modified Quadratic	0.0001	0.3252	0.9843	0.9444	0.8130	20.413

#### Optimization of encapsulation efficiency (EE%)

In general, the most common technique for preparing the CS-based nanocarriers is the ionic-gelation method which was used in the present study for NP preparation. In this process, NPs are made due to electrostatic interacting of the TPP’s anionic agent (PO_4_^−^) and CS’s positive moiety which is created by protonation of –NH_2_ to –NH_3_^+^ in an acidic solution. The optimized amounts of CS, CS:TPP ratio, and TMX:CS ratio were selected pursuant to the EE% of TMX obtained from the DOE software (13.1–62.1%). The EE% value indicates the variation of results concerning the assumed variables (Table 2). According to a central composite statistical design, it was shown that all values for 16 runs were fitted to first order, second order, quadratic, and modified quadratic models (Table 4). The *P* values of the modified quadratic model and lack of fit were 0.0001 (significant) and 0.2784 (not significant), respectively, and R^2^ was 0.8945 (close to 1). The later model showed to be the best one compared to the corresponding results from the first order, second-order, and quadratic models.

#### Selection of optimum formulation for coating with HA

Loading a higher drug amount in the NPs causes delivering more drug to the tumor cells and increases the therapeutic effect that leads to the maximized NPs’ EE% value. Since lowering the particle size positively affects the enhanced permeability and retention (EPR), the NPs’ size was preferred to be minimized. Generally, the optimized condition for the particles of a cancerous tissue-targeting drug-delivery system is a size of 100–200 nm with the maximum EE%. For achieving the optimized condition, we used the modified quadratic model and obtained 1.13, 9, and 0.5 values for the optimal amount of CS concentration, CS:TPP ratio, and TMX:CS ratio, respectively. Also, 153.4.4 nm and 62.21% were predicted for the optimum particle size and EE%, respectively, while they were experimentally measured as 164 ± 7.3 nm and 60 ± 2.2%, respectively. The errors obtained to be <4.1% for particle size and <2.89% for EE%. The same optimization formulation was applied for the HA-coating of nanoparticles.

### Coating of nanoparticles with hyaluronic acid

#### Chemical structure studies by FTIR

**Fig. 1:**
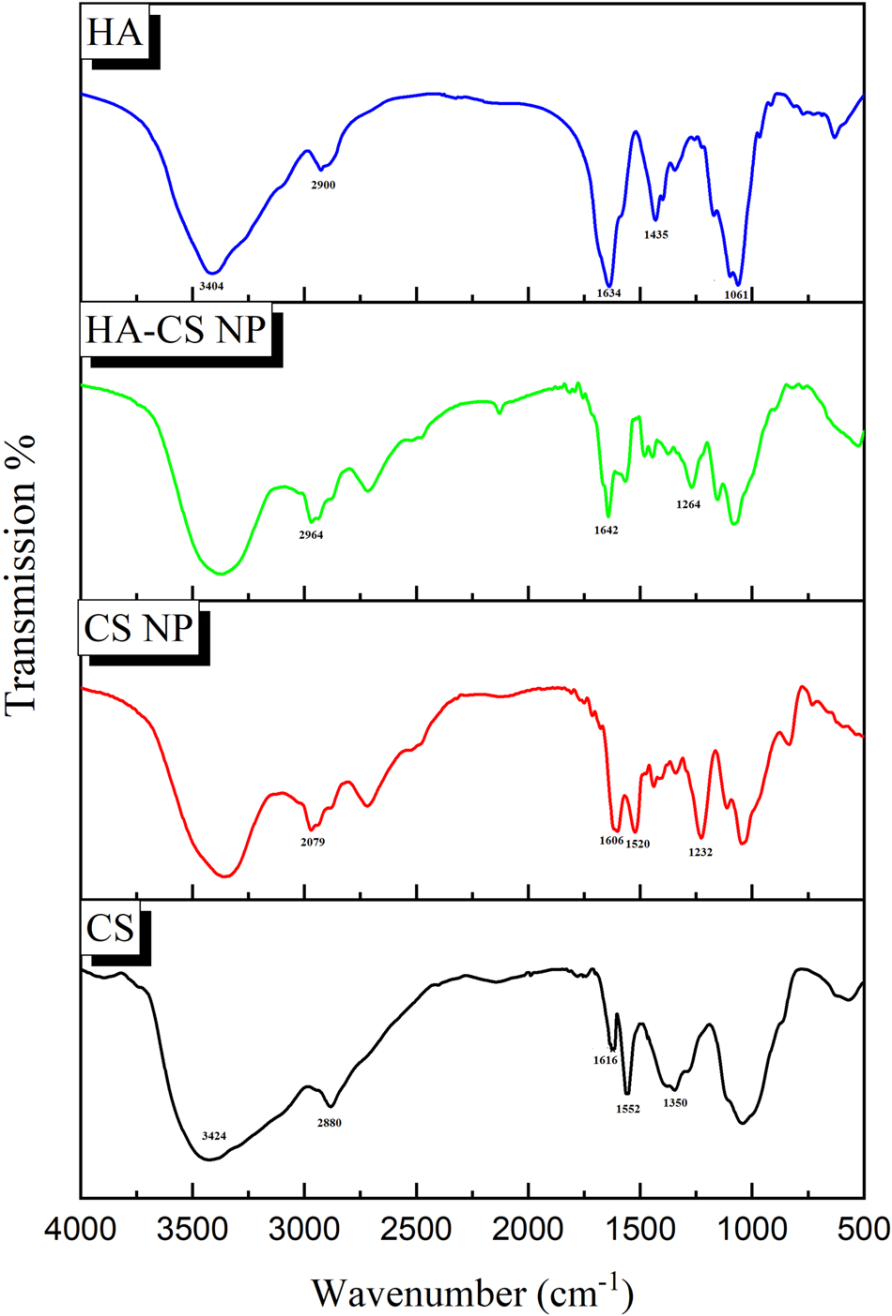
FTIR spectra of chitosan (CS), chitosan nanoparticle (CS NP), hyaluronic acid-coated-chitosan nanoparticle (HA-CS NP), and hyaluronic acid (HA)

The present study aimed to develop a drug-delivery system designed to target specific cancer cell lines using TMX-loaded HA-CS NPs. Accordingly, EDC was used to facilitate attaching the HA carboxylic groups to the CS amino groups to coat CS NPs with the HA. As was mentioned before, the HA coating of NPs enhances their attachment to some types of targeted tissues. For analyzing the linkage between HA and CS NPs, HA, CS, CS NPs, and HA-CS NPs were analyzed by FTIR test (Fig. 1). Accordingly, HA peaks appeared in five points of 3404 cm^−1^ (–OH stretching), 2900 cm^−1^ (–CH stretching), 1634 cm^−1^ (C=O–NH stretching), 1435 cm^−1^ (C–O and C=O), and 1061 cm^−1^ (C–O–C stretching) similar to a previous study [17]. The FTIR spectra of CS also showed five peaks at 3424 cm^−1^ (–OH stretching), 2880 cm^−1^ (C–H stretching), 1616 cm^−1^ and 1552 cm^−1^ (–NH_2_ stretching), and 1350 cm^−1^ (–CH stretching) [35, 36]. The cross-link between CS and TPP caused the peak of 3424 cm^−1^ in CS NP to be less wide and shifted to the right. Besides, the peak of the tensile oscillation of amino groups is in the same range. The peak of CS axial deformation (2880 cm^−1^) is shifted to 2972 cm^−1^ and 2964 cm^−1^ in CS NP and HA-CS NPs, respectively. The bands at 1616 cm^−1^ and 1552 cm^−1^ shifted to 1606 cm^−1^ and 1520 cm^−1^, respectively, which confirm the link between phosphate and amino groups. Also, the peaks of 1232 cm^−1^ (CS NP) and 1264 cm^−1^ (HA-CS NPs) relate to the TPP phosphate groups (P=O and P–O) that indicate the linkage between these negatively charged groups in TPP to the positively charged amino agents of CS. The peaks at 1564 cm^−1^ and 1642 cm^−1^ in the HA-CS NPs spectrum represent the first and second-type amides that approve the linkage between HA and CS amino groups. On the other hand, any peak between 1030 cm^−1^ and 1070 cm^−1^ is considered to be representing primary alcohol [17, 36, 37].

#### Zeta potential and particles size of HA-coated CS nanoparticles

**Fig. 2:**
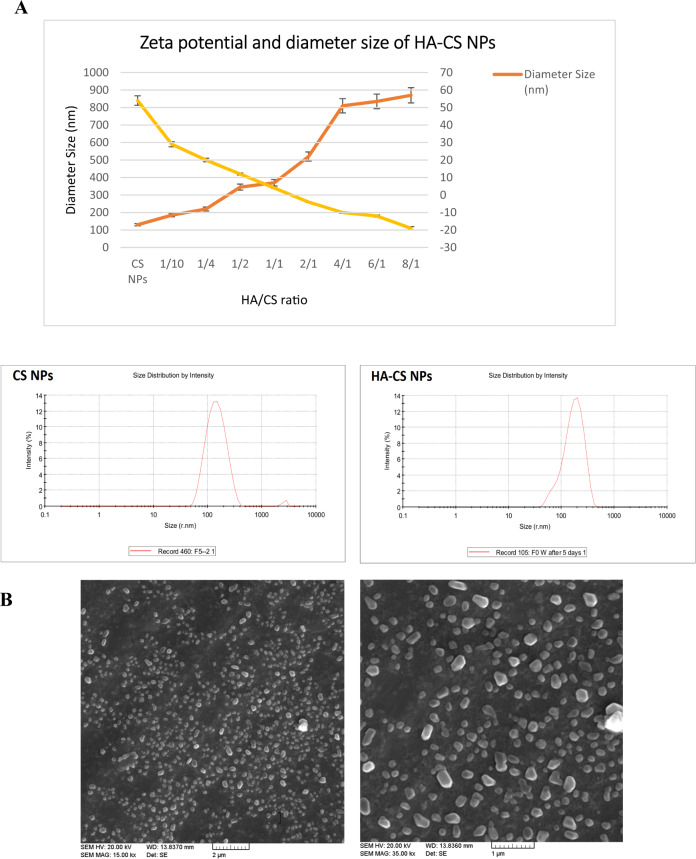
Zeta potential and diameter size of HA-CS NP at different ratios of HA:CS NPs (**A**) and SEM of HA-CS NPs (**B**)

The negative charge of HA cause changing the zeta potential of HA-coated CS NPs. As HA coating reduces the positive charge of CS nanoparticles. Figure 2 represents the result of variations in the particle size and zeta potential by changing the HA:CS ratio. According to this figure, the 1:1 ratio of HA to CS leads to the zeta potential of +17.4 mv and a particle size of 337.1 nm in lack of EDC, while the presence of EDC changes these values to +3.39 mv and 1524 nm, respectively. Therefore, EDC increases the attachment of HA to the surface of CS NPs, acting as a coupling agent between CS and HA. According to the literature, less amount of HA or increased CS NP in the CS NP:HA ratio results in decreasing the particle size and increasing the zeta potential. Since nanoparticles with sizes below 200 nm are shown to be more effective against cancerous cells, different ratios of HA to CS NP have been applied to minimize the particle size to <200 nm. In NPs with the HA:CS ratio of 1:10, the particle size and zeta potential showed to be 180 nm and +20 mv (PDI = 0.212). The optimal zeta potential of nanoparticles prepared in the previous step was +38.4 mv. SEM analyses showed no aggregation of TMX-loaded HA-CS NPs and a spherical and homogeneous shape for these NPs at this ratio (Fig. 2). Generally, adsorption of poly-anions (e.g., HA) on positively charged NPs (e.g., CS) can produce aggregates and/or coagulates probably due to electrostatic interactions or lack of electrostatic stabilization. The size of the NPs and concentrations of polyanion (here HA) and polycation (here CS) impact the aggregation level, so that bigger particles are more likely to aggregate. Considering the SEM results of the present study, the HA:CS ratio of 1:10 can ideally prevent NPs from aggregation (Fig. 2). Moreover, the zeta potential of NPs shows their surface charge and essentially impacts the surface absorption of particles in the serum which determine their clearance of NPs and thereby the NP circulation half-life. On the other hand, positively charged NPs show more affinity to the serum proteins such as fibronectin and laminin helping more rapidly clearance of circulating nano-based drug carrier systems by macrophages located in the liver and spleen within a few hours. Explaining this phenomenon, the positively charged groups on the surface of NPs are suggested to electrostatically interact with the negatively charged groups (e.g., sialic acids and phospholipid head groups) on the surface of macrophages that facilitate the internalization of NPs. Therefore, the NPs’ zeta potential is effectively used for evaluating the efficiency and mechanism of cellular uptake, and the in vivo fate of NPs. Accordingly, low zeta potential or surface charge means less macrophage uptake of NPs. Therefore, HA is used in the carrier formulations because it generally has a negatively charged surface that decreases particle clearance through the reticuloendothelial system (RES) of macrophages. During the present study, the CS NPs zeta potential decreased from +48.9 mv to +26.4 mv through HA coating with an optimized ratio. Therefore, HA coating is not only useful for targeting cancer tissues but also for improving the blood compatibility of particles by reducing their undesirable clearance from the serum. All together, HA coating provides a more efficient drug-delivery procedure to the cancerous tissue.

### In vitroTMX release in different pHs

**Fig. 3:**
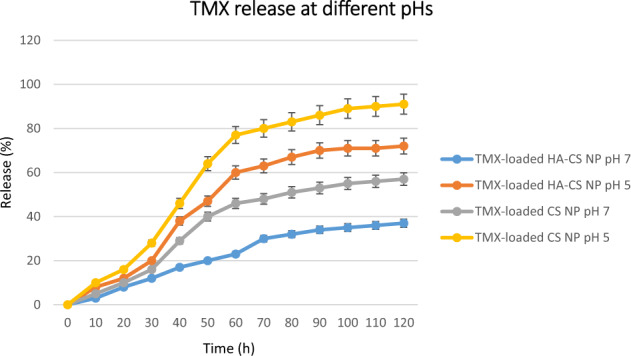
In vitro TMX release from CS NPs and HA-CS NPs at different pHs

The different pH of tumor tissue microenvironment [5, 6] with that of normal tissue (7.4) has led to the design a targeted delivery system containing pH-sensitive components with minimal drug release in physiological pH. These systems are hypothesized to provide more bioavailability of the drug in the tumor site in comparison with the normal tissue and also decrease the side effects of the drug. In the present study, for evaluating the effect of HA on such potential, TMX-loaded CS NPs and TMX-loaded HA-CS NPs were examined for their TMX release potential at both pHs 7.4 and 5 (Fig. 3). According to the present observations, TMX release from both CS NPs and HA-CS NPs was more in acidic pH than in physiologic pH. TMX release from CS NPs was 44% and 82% at pHs 7.4 and 5, respectively. The TMX release from the HA-CS NPs in acidic pH was also higher than in physiologic pH (65%); however, it was lower than that of CS NPs. The facilitated release of drug in the acidic environment is discussed to be due to the break-down of the electrostatic /1‘ balance between CS and TPP in CS NPs, or among CS, TPP, and HA in HA-CS NPs that are formed at pH 7.4. Breaking down the results of the electrostatic interactions in separating the two polymers that lead to so increased drug release. Also, the greater diameter of HA-coated CS NPs increases their resistance to the effects of environmental conditions. Therefore, compared to the CS NPs, the TMX release rate from the HA-CS NPs was 10% and 15% lower in the acidic and physiologic pHs, respectively. This pH-dependent release manner also minimizes the systemic toxicity of the drug due to supplying less bioavailability in healthy organs of the body with physiological pH which in turn reduces the side effects of the drug for the patient. Whereas, this pH-sensitive drug-delivery system provides more bioavailability for the drug in the tumor sites with acidic pHs and confer more drug efficiency in malignant tissues.

### MTT assay

**Fig. 4:**
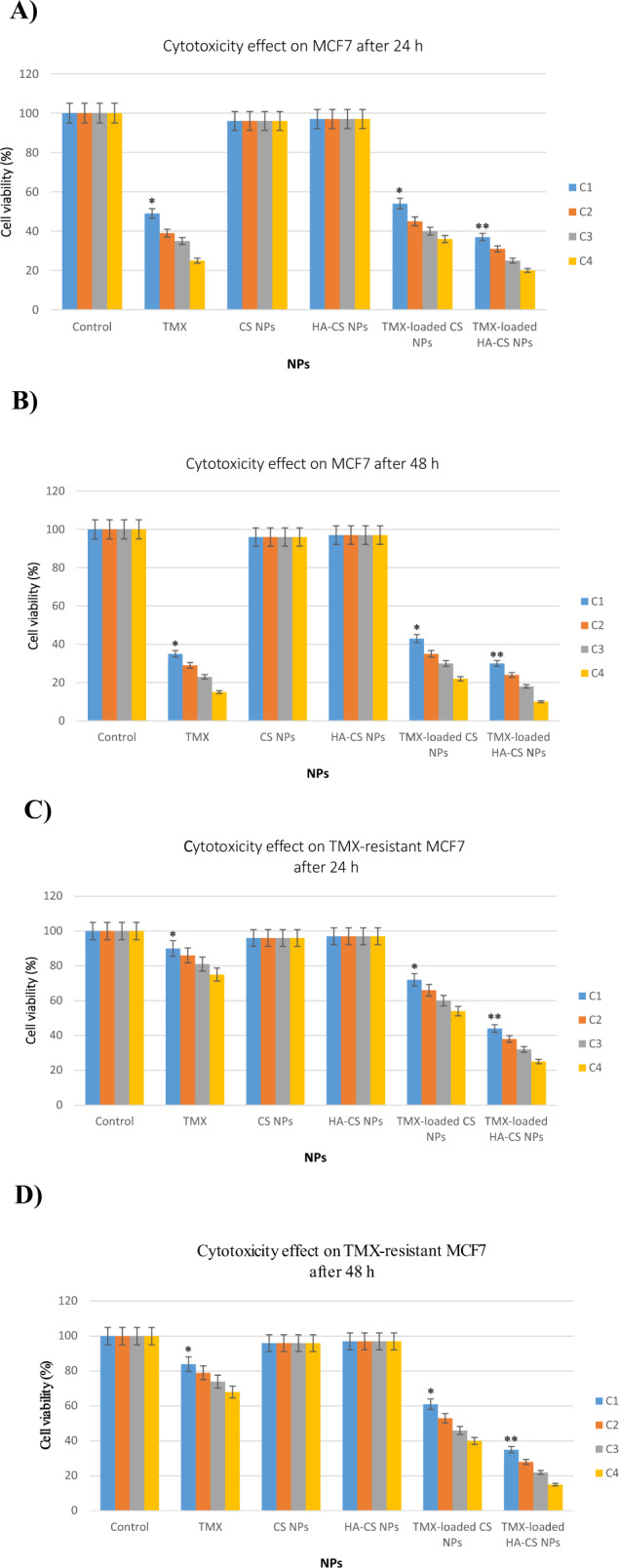
Cytotoxicity of free TMX, CS NPs, HA-CS NPs, TMX-loaded CS NPs, and TMX-loaded HA-CS NPs against MCF7 (**A**, **B**) and TMX-resistant MCF7 (**C**, **D**) cell lines after 24 and 48 h at different concentrations (C1: 3 µg/ml; C2: 6 µg/ml; C3: 12 µg/ml; C4: 24 µg/ml) using MTT assay. *P < 0.05, **P < 0.001, as assessed by Student’s *t* test or one-way ANOVA followed by Tukey *post hoc* test (comparing between more than two groups)

MTT assay was used for examining and comparing the anti-cancer potential of the free TMX, CS NPs, HA-CS NPs, TMX-loaded CS NPs, and TMX-loaded HA-CS NPson both normal cell (HF) and cancer cell lines (MCF7 and TMX-resistant MCF7) within 24 and 48 h (Fig. 4). The concentration of free drug used in this assay was similar to that of all NP’s formulations (Table 3). The drug-free-NP-treated cells showed >95% cell viability at all concentrations of CS NPs and HA-CS NPs after time periods. This observation shows the adequate biocompatibility of drug-free-NPs (CS and HA-CS NPs) and their non-toxicity against all applied cell lines (Fig. 4). Contrarily, the cells that had received drugs - either as free or loaded on NPS (i.e., TMX, TMX-loaded CS NPs, and TMX-loaded HA-CS NPs) - showed a dose-dependent sensitivity to the drug. In addition, the used cells showed the most sensitivity to the TMX-loaded HA-CS NPs compared to the TMX (*P* < 0.05) and TMX-loaded CS NPs (*P* < 0.001). Therefore, the cell survival at the highest concentration of the TMX-loaded HA-CS NPs after 24 h showed to be 20% for the MCF7 cell line (Fig. 4) and 25% for TMX-resistant MCF7 (Fig. 4) cell line. The cell viability of MCF7 and TMX-resistant MCF7 cells decreased by 10% and 15% after 48 h, respectively. Besides, the cell viability of MCF7 and TMX-resistant MCF7 treated with free TMXafter 48 h was about 25% and 70%, respectively. According to the present results, the cytotoxic effects of the same concentrations of free TMX and TMX-loaded HA-CS NPs significantly differ from each other (*P* < 0.05). In conclusion, the less cytotoxic effect of the free TMX compared to the TMX-loaded HA-CS NPs is suggested to be due to the HA coating of the TMX-loaded CS NPs that interacts with the CD44 antigens and more conveniently adsorbed to the receptors that mediate the endocytosis. After 48 h, MTT assay showed free TMX to be more toxic against sensitive MCF7 cells (70%) than the TMX-resistant MCF7 cells (25%). MTT assay also displayed free TMX and TMX-loaded HA-CS NPs to be not toxic to the HLF cells.

### Apoptosis assay

**Fig. 5:**
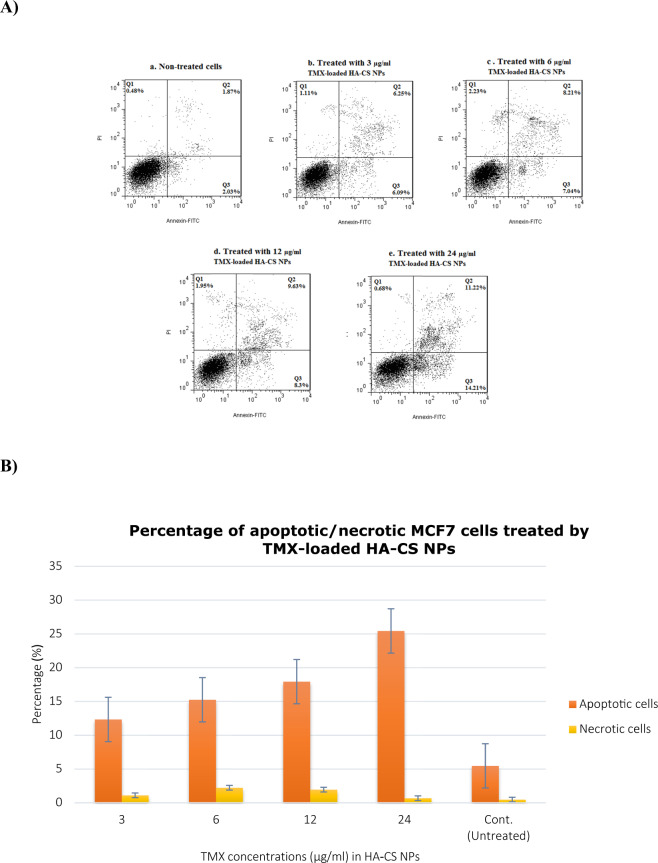
Contour diagram of annexin V/PI flow cytometry. **A** MCF7 cells treated with different concentrations of the TMX (3, 6, 12, & 24 µg/ml) in HA-CS NPs. (a) Untreated cells, (b) MCF7 cells treated with 3 µg/ml TMX loaded in HA-CS NPs, (c) MCF7 cells treated with 6 µg/ml TMX loaded in HA-CS NPs, (d) MCF7 cells treated with 12 µg/ml TMX loaded in HA-CS NPs, (e) MCF7cells treated with 24 µg/ml TMX loaded in HA-CS NPs. Lower left quadrants show viable cells. The upper left quadrants contain the nonviable, necrotic cells. Lower right quadrants represent the apoptotic cells in the early stage. The upper right quadrants represent the apoptotic cells in the late stage. **B** Percentage of apoptotic (in early and late stages)/necrotic MCF7cells treated by TMX-loaded HA-CS NPs at different concentrations of the TMX. *P < 0.05, **P < 0.01, as assessed by Student’s *t* test or one-way ANOVA followed by Tukey post hoc test (comparing between more than two groups)

The apoptotic and necrotic effects of TMX-loaded NPs on the three cell lines were studied using flow cytometry (Fig. 5). According to the results, exposing cells to TMX-loaded HA-CS NPs could considerably induce late apoptosis compared to the control cells in a dose-dependent pattern. Additionally, the cells’ apoptosis level was evaluated as 12.34, 15.25, 17.93, and 25.43% in the presence of 3, 6, 12, 24 µg/ml of TMX-loaded HA-CS NPs, respectively (Fig. 5A, B). At the same time, the cell necrosis level was evaluated as 1.11, 2.23, 1.95, and 0.68%, for the drug-delivery system with the above-mentioned concentrations, respectively. Also, the cells’ apoptosis and necrosis level were obtained as 3.90 and 0.48% for non-treated cells. As shown in Fig. 5, comparing the treated and control cells, the TMX in its highest concentration caused the most apoptosis level (*P* value < 0.001). The rate of apoptotic cells was determined as following: %Apoptosis = %Annexin-V-positive cells + %Annexin-V-positive and PI-positive cells/total cells × 100.

## Discussion

Tamoxifen is a hydrophobic anti-cancer agent approved by the FDA for cancer hormone therapy as a selective estrogen modulator (SERM). Despite its significant efficacy to treat breast cancer, concerns about TAX dose-dependent carcinogenesis persist which limits its therapeutic applications. Nanotechnology can be one of the most important strategies for solving the toxic effect of cancer drugs such as TAX, due to its ability in formulations to deliver low concentrations of drug to cancer cells over a longer period of time [30]. On the other hand, traditional chemotherapy drugs are non-selective and can migrate into almost any part of the body through the bloodstream, interfering with the synthesis of cellular DNA, which lead to the simultaneous death of cancerous and healthy cells that divide rapidly [38]. In some cases, an even higher risk of developing a second primary non-breast cancer has been reported in elderly patients receiving chemotherapy [30]. Therefore, targeted therapy is an ideal treatment option that will inherently lead to fewer complications outside the target area. In addition, one of the most important requirements of nanoformulation suitable for use in the delivery of cancer drugs is the molecular scale. For nanoparticles with a size of 100–800 nm, it allows them to use the tumor environment for selective drug delivery via the enhanced permeability and retention (EPR) effect [39]. Explaining this process, as tumor cells grow rapidly, their need for oxygen and nutrients increases rapidly, which leads to the production of new cancer blood vessels with abnormal architectures. These tumor vessels are made up of poorly aligned endothelial cells with large gaps (about 100–800 nm). In addition, due to the lack of lymphatic drainage, these nanocarriers can persist in tumor tissues for days or even weeks, allowing drugs to be released from the carriers in a sufficient time. This potential, along with modification of nanostructures for specific targeting of tumors, can enhance the process of fighting cancer cells and reduce the severity of drug side effects [30, 40]. Accordingly, in the current study, we evaluated chitosan-based nanoparticles with the aim of targeted delivery of the TMX to breast cancer cells via nanoparticle coating by hyaluronic acid.

The most commonly used nanoparticles in cancer drug delivery, especially for TMX, are polymeric-based NPs (PNPs) which are fabricated primarily from chitosan, starch, and poly (D,L-lactic-co-glycolide (PLGA) [30]. These nanocarriers exhibit excellent safety profiles, biodegradability, biocompatibility, cost-effectiveness, and good capacity for the delivery of hydrophobic and hydrophilic drugs. In addition, for designing a suitable delivery system, the parameters such as size and size distribution, the capacity of drug loading, and stability are the important parameters which are the advantages of polymer-based NPs [41].

Based on studies, by encapsulating TMX into PNPs, the drug molecule is stabilized by hydrogen bonding and hydrophobic interactions within the nanoparticle to form stable drug-polymer compounds that protect the drug from degradation. Based on molecular modeling, the participation of NH_2_ and OH groups in the polymer/drug chemical structure can lead to drug stability in PNPs [42, 43]. According to the studies, TAX-loaded PNPs are suitable for breast cancer treatment because they are able to obtain high drug loading with sustained-release kinetics and high cellular uptake by MCF-7 cell lines in vitro [16, 42]. For example, in a study by Ravikumar et al., the antitumor efficacy of TAX was improved against MCF-7 cell lines by the fabrication of TAX-loaded- PLGA-NPs. Based on their report, TAM-loaded NPs containing 15 mg TAX (highest concentration used) exhibited a particle size of 282.7 nm with a zeta potential of +4 mv and 76.4% encapsulation efficiency. In the current study, the HA-CS particle size was 210 nm with a zeta potential of +25 mv, and the encapsulation efficiency of 55%. It should be noted, zeta potential is an index of nanoparticles stability. In most cases, with increasing the zeta potential of NPs, the amount of surface charge also increases which leads to strong repulsive interaction among the NPs, thus resulting in higher stability and more uniform size of the NPs [44]. Although, studies have shown that chitosan nanoparticles with positive zeta potentials could be cleared from the blood very quickly as compared to the nanoparticle with negative zeta potentials. In both studies, MCF-7 cells were more sensitive to tamoxifen-loaded NPs than tamoxifen alone [45]. However, HA-CS nanoparticles were more effective than PLGA nanoparticles, which could be due to the specific targeting of nanoparticles against MCF-7 cells. Similar to this study, Ravikumara and Madhusudhan, investigated chitosan nanoparticles for tamoxifen delivery and its cytotoxicity against MCF-7 cells [44]. The optimized TMX-loaded CS NPs showed a mean diameter of 187 nm, the zeta potential of +19.1 mv [44]. Similar to the results of this study, TMX-loaded CS NP was significantly more effective against cancer cells than free TMX. As previously noted, they concluded that this difference in efficacy may be due to the enhanced intracellular drug accumulation by nanoparticle uptake. On the other hand, all results confirmed that the cytotoxic effect of nanoparticles is dependent on the time and dose of TMX.

Consistent with this research, a study has been done by Paswan et al., to development of TMX-loaded HA-PLGA nanoparticles for MCF-7 targeting by attaching HA as a ligand to actively target the CD44 receptors present at breast cancer cells surface [46]. The particle size and drug encapsulation efficiency of optimized nanoparticles were 294.8 and 65.16%, respectively. MTT cell line assay showed 47.48% cell mortality when treated with tamoxifen-loaded PLGA- PEG-HA nanoparticles, while the cell survival at the highest concentration of the TMX-loaded HA-CS NPs after 24 h showed to be 20% for the MCF7 cell line and 25% for TMX-resistant MCF7, which shows that TMX-loaded HA-CS nanoparticles are more effective than TMX-loaded HA-PLGA NPs. These results demonstrated that tamoxifen nanoparticles coating with HA are more cytotoxic than tamoxifen drug alone, which is attributed to their preferential uptake by MCF-7 cell lines by the affinity of CD44 receptors of these cells to HA ligand present in nanoparticles.

## Conclusion

Administration of drugs at the required amount, at the requited site, and at the required time in the body reduces the systemic side effects, dosage, and enhances its therapeutic effectiveness [20]. In this regard, the purpose of the current study was the fabrication of CS nanoparticles coated with hyaluronic acid and its application in the field of targeted-tamoxifen delivery. Our findings showed that the tamoxifen loaded in the fabricated composition has a significant suppressive impact in the cancerous cells with lower dosage compared to free drug. The most obvious finding to emerge from this study demonstrated that the fabricated composition can be used as an effective treatment for breast cancer by specific targeting of cancerous cells. However, further research needs to be done to determine the limitations of the release system and the amount of tamoxifen as an effective treatment dose in vivo.

## Data Availability

We confirmed that after publication, the data and associated protocols become promptly available to readers without undue qualifications in material transfer agreements.
